# A bi-functional IL-6-HaloTag^®^ as a tool to measure the cell-surface expression of recombinant odorant receptors and to facilitate their activity quantification

**DOI:** 10.14440/jbm.2017.207

**Published:** 2017-12-15

**Authors:** Franziska Noe, Christiane Geithe, Julia Fiedler, Dietmar Krautwurst

**Affiliations:** Leibniz Institute for Food Systems Biology at the Technical University of Munich, D-85354 Freising, Germany

**Keywords:** De-orphaning, G protein-coupled receptor, GPCR, HEK-293, NxG 108CC15

## Abstract

The functional cell surface expression of recombinant odorant receptors typically has been investigated by expressing N-terminally extended, “tagged” receptors in test cell systems, using antibody-based immunocytochemistry or flow cytometry, and by measuring odorant/receptor-induced cAMP signaling, mostly by an odorant/receptor-induced and cAMP signaling-dependent transcriptional activation of a luciferase-based luminescence assay. In the present protocol, we explain a method to measure the cell-surface expression and signaling of recombinant odorant receptors carrying a bi-functional, N-terminal ‘IL-6-HaloTag^®^’. IL-6, being a secreted cytokine, facilitates functional cell surface expression of recombinant HaloTag^®^-odorant receptors, and the HaloTag^®^ protein serves as a highly specific acceptor for cell-impermeant or cell-permeant, fluorophore-coupled ligands, which enable the quantification of odorant receptor expression by antibody-independent, chemical live-cell staining and flow cytometry. Here, we describe how to measure the cell surface expression of recombinant IL-6-HaloTag^®^-odorant receptors in HEK-293 cells or NxG 108CC15 cells, by live-cell staining and flow cytometry, and how to measure an odorant-induced activation of these receptors by the fast, real-time, luminescence-based GloSensor^®^ cAMP assay.

## BACKGROUND

Odorant receptors (ORs) are seven-transmembrane G protein-coupled receptors (GPCRs) [1], and are activated by small volatile compounds [2,3]. The activation of ORs by odorants triggers a cAMP signaling cascade [4], which activates cellular function of olfactory sensory neurons (OSNs) of the nose [5], ultimately enabling the perception, recognition and discrimination of a plethora of volatile stimuli [6,7].

To investigate the molecular mechanisms of human olfactory perception at the receptor level, cell-based bioassays are required that can cope with (1) complexity of odorant/receptor interactions [8], and (2) individually different and sub-optimal cell surface expression of ORs [9-11]. In the last two decades, different test cell systems have been developed, which facilitated the process of identification of cognate OR/odorant combinations [2,12-15]. The most widely used assay in the recent years has been introduced by the Matsunami group in 2008 [15], a static end point assay, where an activation of OR by odorant results in cAMP signaling, which in turn triggers a cAMP/CRE-dependent transcription of a luciferase transgene, enabling cAMP-proportional luminescence measurements. Most recently, we introduced an alternative, luminescence-based, fast online assay, where a genetically modified, cAMP-binding luciferase [16] (GloSensor^®^, Promega) detects odorant/OR-induced changes in intracellular cAMP, enabling a robust luminescence readout within minutes upon stimulation with odorants [12].

The functional cell-surface expression of recombinant ORs in heterologous cells has been, and until now still is, a major challenge in the field. The identification and co-expression of appropriate signal transducing guanine nucleotide-binding protein G(olf) subunit alpha (Gαolf, GNAL) [4,13], as well as co-expression of accessory proteins and chaperones [11,15], such as receptor transport protein (RTP1S), and the use of N-terminal epitope tags, such as the N-terminal part of rhodopsin (Rho-tag) [2], have largely improved the functional cell surface expression of recombinant ORs in test cell systems, and facilitated their de-orphaning. So far, the most widely used combination of Gαolf, RTP1S, and Rho-tag proved necessary and synergistic for the functional expression of recombinant ORs in test cell systems [11,12].

Recently, a protein epitope tag technology, the so called ‘HaloTag^®^’ (Promega), has been introduced [17]. The bacterial HaloTag^®^ protein warrants little background in mammalian test cell systems, and covalently binds membrane-permeable or impermeable, fluorophore-coupled ligands, which enable antibody-independent live-cell staining and quantification of recombinant HaloTag^®^-OR expression at the cell surface of test cells *via* flow cytometry.

In the present protocol we use HaloTag^®^-ORs that are N-terminally extended by yet another tag, the interleukin 6 (IL-6), a cytokine secreted by immune cells [18] and other cells, which in our hands facilitated the cell surface expression of HaloTag^®^-ORs (see ref [19], **Fig. 3A** and **3B**).

Here, we describe a method how to (1) express recombinant IL-6-HaloTag^®^-ORs in the neuroblastoma X glioma hybrid cell line NxG 108CC15 [20], (2) measure cell surface expression of fluorophore-labelled IL-6-HaloTag^®^-ORs by flow cytometry, and (3) measure odorant/OR-induced cAMP signaling in these cells, using the GloSensor^®^ assay.

## MATERIALS

### Reagents

- Dulbecco’s MEM medium (Cat. #F0435; Biochrom, Berlin, Germany)
- Fetal bovine serum (FBS) superior (Cat. #S0615; Biochrom, Berlin, Germany)
- Penicillin (10000 U/ml)/streptomycin (10000 U/ml) (Cat. #A2212; Biochrom, Berlin, Germany)
- Gibco^®^ HAT supplement (HAT supplement includes hypoxanthine, aminopterin and thymidine) (Cat. #21060-017; Thermo Fisher, Dreieich, Germany)
- MEM non-essential amino acid solution (100 ×) (Cat. #M7145; Sigma-Aldrich, Steinheim, Germany)
- L-glutamine (Cat. #K0282; Biochrom, Berlin, Germany)
- Trypsin-EDTA (Cat. #L2143; Biochrom, Berlin, Germany)
- D-glucose (Cat. #101174Y; VWR Chemicals BDH Prolabo, Leuven, Belgium)
- HEPES (Cat. #441476L; VWR Chemicals BDH Prolabo, Leuven, Belgium)
- NaCl (Cat. #1064041000; Merck, Darmstadt, Germany)
- CaCl_2_*2H_2_O (Cat. #22322.295; VWR Chemicals BDH Prolabo, Leuven, Belgium)
- KCl (Cat. #26764.230; VWR Chemicals BDH Prolabo, Leuven, Belgium)
- NaOH (Cat. #28244.295; VWR Chemicals BDH Prolabo, Leuven, Belgium)
- Dimethyl sulfoxide (DMSO) (Cat. #83673.230; VWR Chemicals BDH Prolabo, Leuven, Belgium)
- Lipofectamine 2000^®^ (Cat. #11668-027; Life Technologies, USA)
- D-luciferin (beetle) monosodium salt (Cat. #E464X; Promega, Madison, USA)
- HaloTag^®^ TMR Ligand (Cat. #G8251; Promega, Madison, USA)
- HaloTag^®^ Alexa Fluor^®^ 488 Ligand (Cat. #G1001; Promega, Madison, USA)
- MACSQuant calibration Beads (Cat. #130-093-607; Miltenyi Biotec GmbH, Bergisch Gladbach, Germany)
- MACSQuant Running Buffer (Cat. #130-092-747; Miltenyi Biotec GmbH, Bergisch Gladbach, Germany)
- MACSQuant Washing Solution (Cat. #130-092-749; Miltenyi Biotec GmbH, Bergisch Gladbach, Germany)
- MACSQuant Storage Solution (Cat. #130-092-748; Miltenyi Biotec GmbH, Bergisch Gladbach, Germany)

### Plasmids

**Figure 1.**
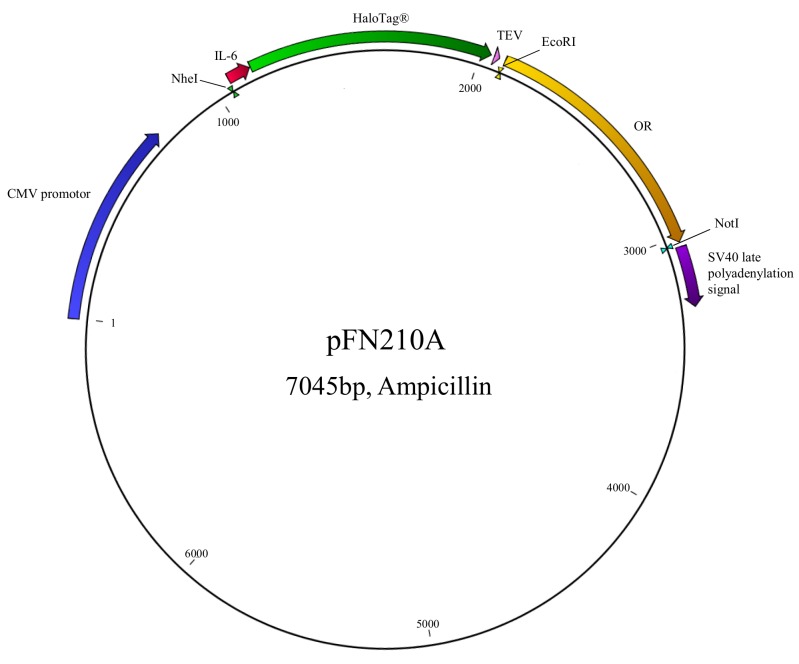
Vector map of expression plasmid pFN210A, carrying the IL-6-HaloTag^®^-OR coding regions. NheI, EcoRI, NotI, recognition sites for restriction endonucleases. TEV (tobacco etch virus) protease recognition sequence. Numbers are base pairs (bp).

- pGloSensor^TM^-22F (Cat. #E2301; Promega, Madison, USA)
- pI2-dk(39aa rho-tag) (aa, amino acid) ([2,13])
- pFN210A (#pFN210A SS-HaloTag^®^ CMV-neo Flexi^®^- Vector, Promega, Madison, USA) (**Fig. 1**)

### Equipment

- 37°C cell culture incubator with 7% carbon dioxide
- 37°C cell culture incubator with 5% carbon dioxide
- Certified class II biological safety cabinet with laminar flow
- Centrifuge universal CT6E (Cat. #521-3605; VWR International GmbH, Darmstadt, Germany)
- High-speed Microcentrifuge CT15E (Cat. #521-3601; VWR International GmbH, Darmstadt, Germany)
- S1000™ Thermal Cycler (Cat. #1852148; BioRad, Muenchen, Germany)
- Nanodrop 2000 (Thermo Fisher Scientific, Waltham, USA)
- White 96-well plates (Cat. #136102; Nunc, Roskilde, Denmark)
- Tissue culture plate, 12 well, flat bottom with low evaporation lid (Cat. #353043; Falcon^®^ Corning Incorporated – Life Sciences, Durham, USA)
- Glomax^®^ MULTI^+^ detection system (Cat. #GM3000; Promega, Madison, USA)
- MACSQuant Analyzer 10 (Cat. #130-096-343; Miltenyi Biotec GmbH, Bergisch Gladbach, Germany)
- Chill 96 Rack (Cat. #130-094-459; Miltenyi Biotec GmbH, Bergisch Gladbach, Germany)

### Recipes

Culture medium: NxG 108CC15 cells [20] are grown in 4.5 g/L D-glucose containing DMEM medium supplemented with 10% FBS, 4 mmol/L L-glutamine, 100 units/ml penicillin, 100 units/ml streptomycin, 100 μmol/L hypoxanthine, 0.4 μmol/L aminopterin, 16 μmol/L thymidine (HAT media supplement), and 1% of 100 MEM nonessential amino acid (NEAA). Stock cells are mounted in Culturing medium containing 11% DMSO.

HEK-293 cells are grown in 4.5 g/L D-glucose containing DMEM medium supplemented with 10% FBS, 2 mmol/L L-glutamine, 100 units/ml penicillin, 100 units/ml streptomycin. Stock cells are mounted in Culturing medium containing 11% DMSO.

Store culturing medium at 4°C and pre-warm it at 36°C before usage.

D-luciferin stock solution: Prepare a 10 mM HEPES solution and adjust to pH 7.5. Dilute 250 mg of D-luciferin (beetle) monosodium salt in 7.5 ml HEPES. Aliquot into 115 μl stock and store them at **–**80°C until use.

Buffer for luminescence measurements: Prepare 1M solutions in distilled water for NaCl, KCl, CaCl_2_ and HEPES. Solutions have to be autoclaved but can be stored at room temperature. Prepare buffer on the day of use, containing 140 mM NaCl, 20 mM HEPES, 5 mM KCl, 1 mM CaCl_2_ and 10 mM D-glucose. Adjust to pH 7.5 by using a 1 M NaOH solution.

### Software

- CLC Main Workbench Software (Cat. #832030; QIAGEN Bioinformatics, Aarhus, Denmark)
- MACSQuantify Software (Cat. #130-094-556; Miltenyi Biotec GmbH, Bergisch Gladbach, Germany)
- SigmaPlot 10.0 (Systat Software GmbH, Erkrath, Germany)

## PROCEDURE

### Preparation of a stock of NxG 108CC15 or HEK-293 cells

1. Rapidly thaw cells.
2. Resuspend the cells with 10 ml of culturing medium and transfer the suspension to a 10 cm cell-culture dish. Culture NxG cells in a 37°C incubator with 7% CO_2_. Culture HEK-293 cells in a 37°C incubator with 5% CO_2_.
3. Check cells under a phase contrast microscope to make sure that the cells are healthy.

**Figure 2.**
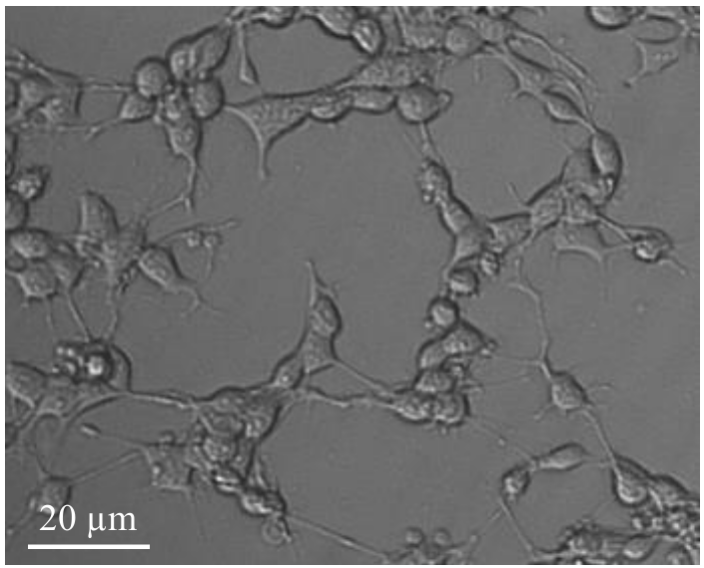
Light-optical microscopy of NxG 108CC15 cells in culture. Microscopic image was taken with a Zeiss Axiovert 25, Zeiss A-Plan 20x/0.3 Ph 1 Var1 and AxioCam MRm.

The cells should be max. 80% confluent (**Fig. 2**). Use the cells between the second and max. tenth cell-passage.

### Transfer cells for transfection

***4.*** Aspirate all medium from the cell culture dish. Transfer 1 ml of trypsin-EDTA solution onto the cells. Gently agitate the dish to allow the cells detaching from the bottom of the dish. Add 9 ml of culturing medium to the dish.
***5.*** Transfer the cell suspension to a 50 ml conical tube and centrifuge for 3 min at 250 *g* at room temperature.
***6.*** Follow option step 6.1 if intending to transfect the cells for analysis of OR activation by the cAMP-luminescence assay. Follow option step 6.2 if intending to transfect the cells for evaluation of cell-surface expression by flow cytometry.
**Note:** Since cells follow gravity, use the pipette to resuspend cells (3 up/down) each time before transferring them to the plate.
  ***6.1.***Preparing cells for transfection in 96-well plates used for measuring the activation of ORs *via* a cAMP-luminescence assay
    ***6.1.1.***For each 96-well plate 7.2 × 10^5^ NxG cells or 11.5 × 10^5^ HEK-293 cells are needed. Re-suspend the desired amount of cells in 10 ml culturing medium and transfer the cell suspension into a reagent reservoir.
    ***6.1.2.***By using a multichannel pipette, distribute 100 μl of the cell suspension from the reservoir into each well of a 96-well plate.
    ***6.1.3.***Culture 96-well plates in the incubator overnight before proceeding with step 7.
  ***6.2.***Preparing cells for transfection in 12-well plates used for measuring cell surface expression
    ***6.2.1.***For each 12-well plate 9.6 × 10^5^ NxG cells or 11.5 × 10^5^ HEK-293 cells are needed. Resuspend the desired amount of cells in 9.6 ml culturing medium.
    ***6.2.2.***Transfer 0.8 ml of the cell suspension into each well of a 12-well plate.
    ***6.2.3.***Culture 12-well plates in the incubator overnight before proceeding with step 7.

### Transfection

***7.*** Transfect cells as described below, depending on experimental setup. For cAMP-luminescence assay follow step 7.1, for flow cytometry follow option step 7.2.
  ***7.1.***Preparing transfection mixture for 96-well plates
    ***7.1.1.***For each well, 25 l serum free medium, and the following amounts of OR constructs or control plasmid without OR-coding region (‘mock’ control), as well as accessory factor constructs (RTP1S, Gαolf, pGloSensor^TM^-22F) are needed (**Table 1**).
    ***7.1.2.***For each well mix 0.625l Lipofectamine 2000^®^ with 25 μl serum free medium in a 1.5 ml microcentrifuge tube and incubate it for 5 min at room temperature.
    ***7.1.3.***Mix the DNA mixture with the Lipofectamine 2000^®^ mixture with a vortex mixer and incubate at room temperature for 18 min.
    ***7.1.4.***Add the 50 μl of the transfection mixture to each well, of the prepared cells in the 96-well plate containing 100 μl culturing medium.
    ***7.1.5.***Culture cells in a 37_°C_ for NxG 108CC15 cells, or 5% CO_2_ for HEK-293 cells, for 42 h before proceeding with step 8.1, but replace medium by 100μl fresh culturing medium within 18 h.
  ***7.2.***Preparing transfection mixture for 12-well plates
    ***7.2.1.***For each well 200 l serum free medium, and the following amounts of OR constructs or control plasmid without OR-coding region (‘mock’ control), as well as accessory factor constructs (RTP1S,olf, pGloSensor^TM^-22F) are needed (**Table 2**).
    ***7.2.2.***For each well mix 5l Lipofectamine 2000^®^ with 200l serum free medium in a 1.5 ml microcentrifuge tube and incubate it for 5 min at room temperature.
    ***7.2.3.***Mix the DNA mixture with the Lipofectamine 2000^®^ mixture with a vortex mixer and incubate at room temperature for 18 min.
    ***7.2.4.***Add 400 μl of the transfection mixture to each well, of the prepared cells in the 12-well plate containing 800 μl culturing medium.
    ***7.2.5.***Culture cells in a 37_°C_ for NxG 108CC15 cells, or 5% CO_2_ for HEK-293 cells, for ~24 h before proceeding with step 8.2, but replace medium by 800 μl fresh culturing medium within 18 h.

### Measurement

**Figure 3.**
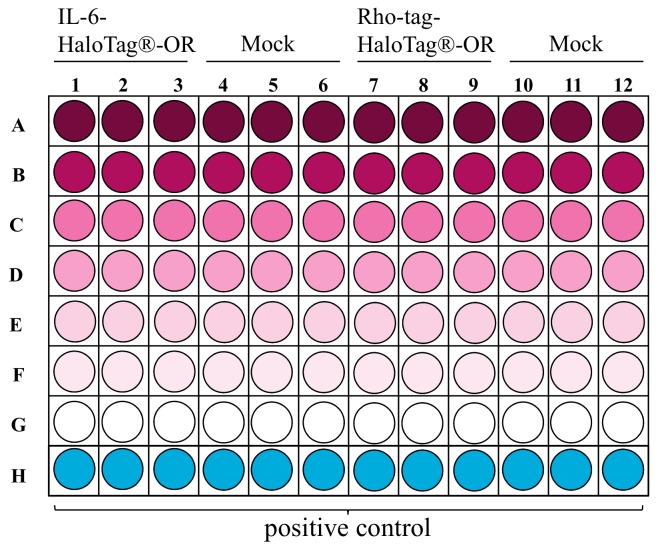
Example layout of a 96-well plate showing the distributions of dilutions for concentration-response relations with two differently tagged ORs. Decreasing concentrations from A-G are color intensity-coded.

***8.*** For measuring the OR activation by the cAMP-luciferase assay follow the instructions of step 8.1. Step 8.2 describes the procedure for measuring the cell-surface expression of ORs by flow cytometry.
  ***8.1.***Measuring activation of ORs *via* a cAMP-luminescence assay
    ***8.1.1.***Preparation of the measurement buffer
    Prepare the buffer as described in the chapter “recipes”.
    ***8.1.2.***Preparation of stock solutions and serial dilutions of odorants
      1. Allow the odorant to achieve room temperature before opening. Add the desired amount of odorant to DMSO. Dissolve the odorant by gentle mixing. For some odorants, it is possible to store the stock solution at **–**20C. Odorants that need to be stored under inert gas (like argon or nitrogen) according to their chemical properties need to be freshly prepared every time before usage.
      2. Dilutions should always be prepared fresh on the day of use. Dilute the odorant stock solution in the prepared buffer. Add 1 μl stock solution to 999 l buffer to obtain a final concentration of DMSO of 0.1%. Serially dilute down the odorant with 0.1% DMSO containing buffer.
    Which concentrations should be used to establish a concentration-response relation depends on each odorant/OR combination. The concentration range in which a GPCR may be fully activated by its agonist, assuming a single type of non-cooperative binding sites, typically spans about 2.5 orders of magnitude, and has to be adjusted according to, for example, information available from single concentrations that yielded ‘hits’ in screening experiments. A limiting factor, however, can be the solubility of odorants to be tested: Many odorants are difficult to dissolve in the solvent/physiological buffer used.
    ***8.1.3.***Measuring the activation of ORs
      1. Prepare the buffer, stock solutions of odorants and perform the serial dilutions as described above.
      2. Aspirate all medium from each well by using an 8-channel adapter.
      3. Thaw an appropriate amount of pre-made luciferin stocks. Prepare the buffer/luciferin mixture by adding one stock luciferin to 6 ml buffer containing 0.1% DMSO for one 96-well plate. Using a multichannel pipette, distribute 60 μl buffer/luciferin mixture to each well.
      4. Incubate the plate at room temperature in the dark for 50 min.
      5. Measure luminescence with the Glomax^®^ Multi^+^ detection system. As ‘basal level’ measure three data points.
      6. Using a multichannel pipette, add 30l of the serial dilution, in case of measuring concentration-response relations, or a single dilution of the desired odorant, in case of measuring receptor screening experiments, to each well (**Fig. 3**).
      7. Incubate the plate at room temperature in the dark for ≥ 4 min.
      8. Measure luminescence with the Glomax^®^ Multi^+^ detection system.
  ***8.2.***Measuring cell surface expression
    ***8.2.1.***Detach cells by using cell scrapers and transfer cell suspension into 1.5 ml microcentrifuge tubes. [Info: Typically, in cell culture trypsin is used to destroy cell/dish surface contacts, and to get adherent cells in suspension. Fortunately, NxG 108CC15 cells are easily detached from cell culture dishes by harvesting them with a cell scraper. Moreover trypsin is omitted to not compromise fluorophore labelling of the extracellular HaloTag^®^ site.] Centrifuge the tubes at room temperature for 8 min at 61 *g*.
    ***8.2.2.***Prepare a 0.5% ‘staining solution’ of either the membrane non-permeable ligand HaloTag^®^ AlexaFluor 488, or the membrane permeable HaloTag^®^ TMR ligand in serum-free medium. Aspirate all medium from the centrifuged cells, retaining the cell pellet at the bottom of the tube. Add 120 μl serum-free medium, as well as 30 μl of the 0.5% staining solution. Re-suspend cells gently.
    ***8.2.3.***Put the tubes into a cell culture dish and incubate for 1 h at 37°C and 7% CO_2_ (NxG 108CC15 cells) or 5% CO_2_ (HEK-293 cells).
    ***8.2.4.***Centrifuge the tubes at room temperature for 8 min at 61 *g*. Aspirate all solution and carefully re-suspend the cells with 400 μl serum free medium. Centrifuge at room temperature for 8 min at 61 *g*. Repeat this step for each sample twice.
    ***8.2.5.***For samples labeled with the HaloTag^®^ AlexaFluor 488 ligand go directly to step 8.2.7. For samples labeled with the membrane permeable HaloTag^®^ TMR ligand re-suspend the cells in 500 μl culturing medium and incubate them for half an hour at 37°C and 7% CO_2_ (NxG 108CC15 cells) or 5% CO_2_ (HEK-293 cells).
    ***8.2.6.***Centrifuge the tubes at room temperature for 8 min at 61 *g*.
    ***8.2.7.***Prior to FACS analyses set the forward- and side-scatter gate. Use for the NxG 108CC15 cells (FSC: 235V and SSC: 360V), and use for AlexaFluor 488-labeled cells the FITC signal (B1-channel 175V) and for TMR labeled cells the PE signal (B2-channel 332V). Use for the HEK-293 cells (FSC: 240V and SSC: 395V) and use for AlexaFluor 488-labeled cells the FITC signal (B1-channel 195V) and for TMR labeled cells the PE signal (B2-channel 332V).Use 10000 cells for each measurement.
    ***8.2.8.***Aspirate all solution and re-suspend the cells with 180 μl serum free medium. Transfer the cell resuspension into a 96-well skirted PCR plate and start flow cytometry analysis.

### Data analysis

***9.*** The data analysis for the cAMP-luminescence assay is performed according to step 9.1, and according to step 9.2 for the flow cytometry assay.
  ***9.1.***Data analysis for the cAMP-luminescence assay
    ***9.1.1.***Primary data analysis
    The raw luminescence data obtained from the Glomax is saved as an Excel worksheet. Three consecutive data point values before (‘basal level’) and after odorant application are each averaged, and the respective averaged ‘basal level’ is subtracted from each signal.
    ***9.1.2.***Secondary data analysis – OR screening experiments
      1. The duplicates get averaged.
      2. To evaluate responding receptors, a 2-sigma (2σ) or 3-sigma (3σ) threshold (mean of all receptor responses + two or three standard deviations) should be calculated.
    ***9.1.3.***Secondary data analysis – non-linear regression
      For concentration-response relations, it is conventional to have at least three independent transfection experiments, each performed in triplicates. Amplitudes should be normalized to those of a defined OR/odorant pair within each experiment. Alternatively, OR amplitudes may be normalized to an endogenous GPCR-induced cAMP luminescence signal.
      1. Average the normalized data of all three experiments.
      2. EC_50_ values and curves are derived from fitting the function.
      f(*x*) = ((min **–** max)/(1 + (*x*/EC_50_)^hillslope^)) + max, or
      f(*x*) = 1/(1 + (EC_50_/*x*)^hillslope^) to the data of a rising concentration-response relation by non-linear regression.
  ***9.2.***Data analysis for the flow cytometer assay
    ***9.2.1.***Primary data analysis
      1. Open the data files with the MACSQuantify Software.
      2. Set the gate for the live cells to exclude dead cells of the further analysis in the 2D plot. The FSC signal (x-axis) is proportional to the surface area of the measured cell whereas the SSC signal (y-axis) depends on the granularity of the cell.
      3. Upon excitation with an argon laser (488 nm), AlexaFluor 488-labelled cells emit green light. Set the gate for the mock-transfected cells at 1% in the 2D plot. The FITC signal of each mock control defines the distinction between negative and positive cells.
      4. TMR-labelled cells show two excitation maxima: one at around 488 nm and one at around 561 nm. The PE molecule is more efficiently excited at 561 nm and has a fluorescence emission peak at 578 nm. The PE signal of each mock-control defines the distinction between negative and positive cells.
      5. Copy both gates for all other investigated samples.

## ANTICIPATED RESULTS

### Cell-surface expression of fluorophore-labelled ORs

**Figure 4.**
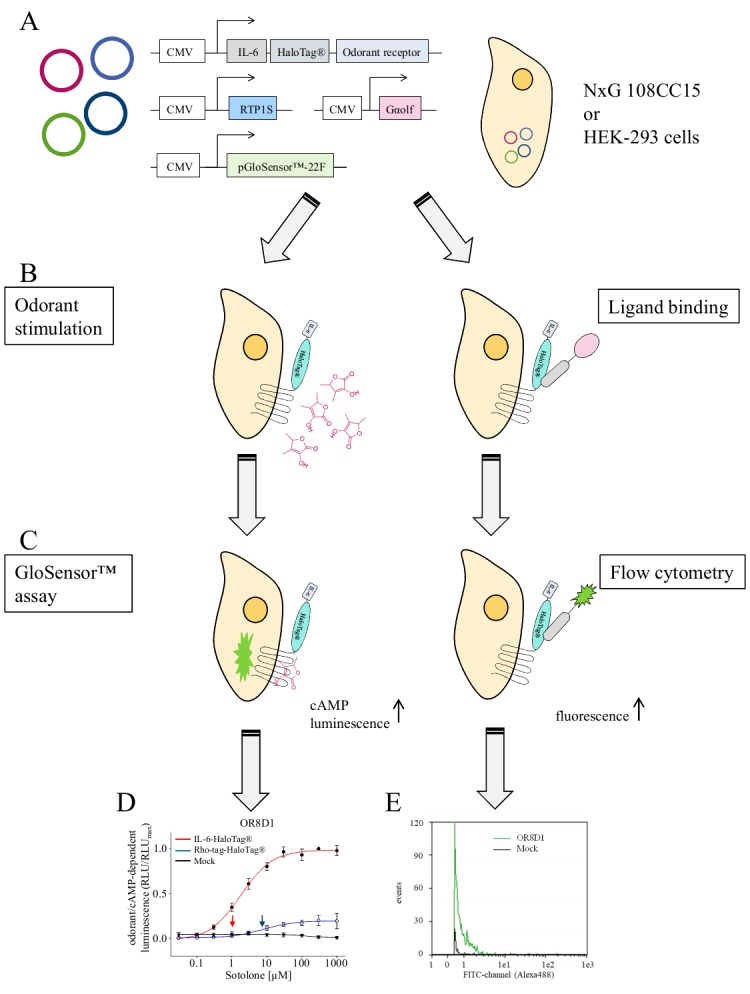
Graphical summary of the methods and examples of expected experimental outcome. **A.** Transfection of cells with expression plasmids coding for a given OR, the chaperone RTP1S, the olfactory G protein alpha subunit, and the cAMP-activated luciferase (pGloSensor™-22F). **B.** Left panel, odorant stimulation of transiently transfected cells. Right panel, Binding of fluorescence-coupled ligand to the HaloTag^®^. **C.** Quantification of odorant-induced, cAMP-dependent luminescence (left panel) by plate reader (**Fig. 2A** in [12]), and of HaloTag^®^/Ligand-dependent fluorescence by flow cytometry (right panel). **D.** Example result of a concentration-response relation of sotolone on OR8D1, equipped with different N-terminal tags. Data are mean ± SD (*n* = 3–5), normalized to the maximum amplitude of OR8D1 carrying the N-terminal IL-6-HaloTag^®^. Red colored curve indicates receptor with the N-terminal IL-6-HaloTag^®^, blue, with Rho-tag-HaloTag^®^, and black curve indicates the empty plasmid control (Mock). Curves were derived from fitting the logistic function to the data. Arrows indicate EC_50_ values (see ref [19], **Fig. 1E**). **E.** Flow cytometry-derived fluorescence distribution of ~1000 NxG 108CC15 cells expressing IL-6-HaloTag^®^-OR8D1, and labeled with cell membrane-impermeant HaloTag^®^ Ligand-Alexa488 (see ref [19] Fig. S3E).

Live-cell flow cytometry delivers at least two important parameters, the number of recorded cells that emitted a receptor-related fluorescence signal, and the fluorescence intensity emitted by each recorded cell (**Fig. 4E**). These two parameters, typically measured from thousands of cells, enable a quantification of OR cell-surface expression. It is conventional to have at least three replicates of the experiment.

### cAMP luminescence assay

ORs are known to have a so-called constitutive or basal activity, which means that to a certain degree they may activate cAMP signaling even in the absence of an adequate odorant stimulus [21]. This basal activity may vary significantly among the different ORs. As a result, in our GloSensor^®^ cAMP assay, the luciferase activity could be significantly different when using different ORs. Therefore, it is important to determine the luminescence background of each well, and to subtract the average luminescence value of each well before application of odorant from the respective averaged luminescence values in the presence of odorant, to obtain baseline-subtracted Δ luminescence values. Each Δ luminescence value obtained from an odorant-activated OR can then be normalized against the maximum Δ luminescence value of a control receptor, or the highest Δ luminescence obtained for that particular OR in this experiment.

For OR screening experiments, all amplitudes above a calculated 3σ-threshold may be considered as signals (“hits”), but have to be validated by establishing concentration-response-relations in subsequent transfection experiments.

For concentration-response-relations, typically any OR-specific and odorant concentration-dependent effects may be fitted by a non-linear regression algorithm, which will deliver the effective concentration values at half-maximal response (EC_50_) (**Fig. 4D**).

It is conventional to have three replica wells and to replicate the experiment three times. The mean relative luminescence unit (RLU), standard deviation and fitted functions can then be graphed with an appropriate software.

## TROUBLESHOOTING

Possible problems and their troubleshooting solutions are listed in **Table 3**.

## Figures and Tables

**Table 1.** Transfection mixture for the cAMP luminescence assay.

Plasmid DNA	Amount (ng)
OR (or: ‘empty’ plasmid = ‘mock’ control)	100
RTP1S	50
Gαolf	50
pGloSensor^™^-22F	50

**Table 2.** Transfection mixture for the flow cytometer assay.

Plasmid DNA	Amount (ng)
OR (or: ‘empty’ plasmid = ‘mock’ control)	800
RTP1S	400
Gαolf	400
pGloSensor^™^-22F	400

**Table 3.** Troubleshooting.

Step	Problem	Cause	Suggestions
8.1	Odorant insoluble	Intrinsic physicochemical properties	Prepare the stock solution in ethanol and also the serial dilution with an addition of 0.1% ethanol to the buffer instead of DMSO Use a detergent like Pluronic PE-10500

## References

[1] Buck L Axel R (1991) A novel multigene family may encode odorant receptors - a molecular-basis for odor recognition. Cell 65: 175-187. doi: 10.1016/0092-8674(91)90418-X. PMID: 1840504

[2] Krautwurst D Yau KW Reed RR (1998) Identification of ligands for olfactory receptors by functional expression of a receptor library. Cell 95: 917-926. doi: 10.1016/S0092-8674(00)81716-X. PMID: 9875846

[3] Zhao H Ivic L Otaki JM Hashimoto M Mikoshiba K (1998) Functional expression of a mammalian odorant receptor. Science 279: 237-242. doi: 10.1126/science.279.5348.237. PMID: 9422698

[4] Reed RR (1992) Signaling pathways in odorant detection. Neuron 8: 205-209. doi: 10.1016/0896-6273(92)90287-N. PMID: 1739458

[5] Touhara K Vosshall LB (2009) Sensing odorants and pheromones with chemosensory receptors. Annu Rev Physiol 71: 307-332. doi: 10.1146/annurev.physiol.010908.163209. PMID: 19575682

[6] Bushdid C Magnasco MO Vosshall LB Keller A (2014) Humans can discriminate more than 1 trillion olfactory stimuli. Science 343: 1370-1372. doi: 10.1126/science.1249168. PMID: 24653035 PMC4483192

[7] Dunkel A Steinhaus M Kotthoff M Nowak B Krautwurst D (2014) Nature's chemical signatures in human olfaction: a foodborne perspective for future biotechnology. Angew Chem Int Ed Engl 53: 7124-7143. doi: 10.1002/anie.201309508. PMID: 24939725

[8] Malnic B Hirono J Sato T Buck LB (1999) Combinatorial receptor codes for odors. Cell 96: 713-723. PMID: 10089886 10.1016/s0092-8674(00)80581-4

[9] Ghiringhelli C Ghiringhelli L Redaelli G Crespi B (1992) [Skin versus mesh in the treatment of laparoceles]. Minerva Chir 47: 419-422. doi: 10.1111/j.1471-4159.2004.02619.x. PMID: 1534152

[10] McClintock TS Sammeta N (2003) Trafficking prerogatives of olfactory receptors. Neuroreport 14: 1547-1552. doi: 10.1097/01.wnr.0000085904.20980.e1. PMID: 14502073

[11] Zhuang H Matsunami H (2007) Synergism of accessory factors in functional expression of mammalian odorant receptors. J Biol Chem 282: 15284-15293. doi: 10.1074/jbc.M700386200. PMID: 17387175

[12] Geithe C Andersen G Malki A Krautwurst D (2015) A Butter Aroma Recombinate Activates Human Class-I Odorant Receptors. J Agric Food Chem 63: 9410-9420. doi: 10.1021/acs.jafc.5b01884. PMID: 26451762

[13] Shirokova E Schmiedeberg K Bedner P Niessen H Willecke K (2004) Identification of specific ligands for orphan olfactory receptors. G protein-dependent agonism and antagonism of odorants. J Biol Chem 280: 11807-11815. doi: 10.1074/jbc.M411508200. PMID: 15598656

[14] Touhara K (2007) Deorphanizing vertebrate olfactory receptors: recent advances in odorant-response assays. Neurochem Int 51: 132-139. doi: 10.1016/j.neuint.2007.05.020. PMID: 17640771

[15] Zhuang H Matsunami H (2008) Evaluating cell-surface expression and measuring activation of mammalian odorant receptors in heterologous cells. Nat Protoc 3: 1402-1413. doi: 10.1038/nprot.2008.120. PMID: 18772867 PMC2664838

[16] Binkowski B Fan F Wood K (2009) Engineered luciferases for molecular sensing in living cells. Curr Opin Biotechnol 20: 14-18. doi: 10.1016/j.copbio.2009.02.013. PMID: 19299118

[17] Urh M Rosenberg M (2012) HaloTag, a Platform Technology for Protein Analysis. Curr Chem Genomics 6: 72-78. doi: 10.2174/1875397301206010072. PMID: 23213345 PMC3480824

[18] Assier E Boissier M Dayer J (2010) Interleukin-6: from identification of the cytokine to development of targeted treatments. Joint Bone Spine 77: 532-536. doi: 10.1016/j.jbspin.2010.07.007. PMID: 20869898

[19] Noe F Frey T Fiedler J Geithe C Nowak B (2017). IL-6–HaloTag^®^ enables live-cell plasma membrane staining, flow cytometry, functional expression, and de-orphaning of recombinant odorant receptors. J Biol Methods 4: e81. doi: 10.14440/jbm.2017.206. PMC6706138 31453235

[20] Hamprecht B Glaser T Reiser G Bayer E Propst F (1985) Culture and characteristics of hormone-responsive neuroblastoma X glioma hybrid cells. Methods Enzymol 109: 316-341. PMID: 2985920 10.1016/0076-6879(85)09096-6

[21] Reisert J (2010) Origin of basal activity in mammalian olfactory receptor neurons. J Gen Physiol 136: 529-540. doi: 10.1085/jgp.201010528. PMID: 20974772 PMC2964517

